# Association of age and spinopelvic function in patients receiving a total hip arthroplasty

**DOI:** 10.1038/s41598-023-29545-5

**Published:** 2023-02-14

**Authors:** Henryk Haffer, Zhouyang Hu, Zhen Wang, Maximilian Müllner, Sebastian Hardt, Matthias Pumberger

**Affiliations:** Center for Musculoskeletal Surgery, Charité - Universitätsmedizin Berlin, Corporate Member of Freie Universität Berlin, Humboldt-Universität zu Berlin, Berlin, Germany

**Keywords:** Medical research, Risk factors

## Abstract

Restricted spinopelvic mobility received attention as a contributing factor for total hip arthroplasty (THA) instability. However, it is still unknown, how the spinopelvic function is influenced by age. In identifying the patients at highest risk for altered spinopelvic mechanics the study aimed to determine the association of age on the individual segments of the spinopelvic complex and global spinal sagittal alignment in patients undergoing THA. 197 patients were included in the prospective observational study conducting biplanar stereoradiography (EOS) in standing and sitting position pre-and postoperatively. Two independent investigators assessed C7-sagittal vertical axis (C7-SVA), cervical lordosis (CL), thoracic kyphosis (TK), lumbar lordosis (LL), pelvic incidence (PI), sacral slope (SS), anterior plane pelvic tilt (APPT), and pelvic femoral angle (PFA). Key segments of the spinopelvic complex are defined as lumbar flexibility (∆ LL = LL_standing_ − LL_sitting_), pelvic mobility (∆ SS = SS_standing_ − SS_sitting_) and hip motion (∆ PFA = PFA_standing_ − PFA_sitting_). Pelvic mobility was further defined based on ∆ SS = SS_standing_ − SS_sitting_ as stiff (∆ SS < 10°), normal (∆ SS ≥ 10°–30°) and hypermobile (∆ SS > 30°). The patient collective was classified into three groups: (1) < 60 years (n = 56), (2) ≥ 60–79 years (n = 112) and (3) ≥ 80 years (n = 29). Lumbar flexibility (∆ LL) was decreased with increasing age between all groups (36.1° vs. 23.1° vs. 17.2°/p_1+2_ < 0.000, p_2+3_ = 0.020, p_1+3_ < 0.000) postoperatively. Pelvic mobility (∆ SS) was decreased in the groups 2 and 3 compared to group 1 (21.0° and 17.9° vs. 27.8°/p_1+2_ < 0.000, p_2+3_ = 0.371, p_1+3_ = 0.001). Pelvic retroversion in standing position (APPT) was higher in group 2 and 3 compared to group 1 (1.9° and − 0.5° vs 6.9°/p_1+2_ < 0.000, p_2+3_ = 0.330, p_1+3_ < 0.000). Global sagittal spinal balance (C7-SVA) showed more imbalance in groups 2 and 3 compared to group 1 (60.4 mm and 71.2 mm vs. 34.5 mm/p_1+2_ < 0.000, p_2+3_ = 0.376, p_1+3_ < 0.000) postoperatively. The preoperative proportion of patients with stiff pelvic mobility in group 1 was distinctly lower than in group 3 (23.2% vs. 35.7%) and declined in group 1 to 1.8% compared to 20.7% in group 3 after THA. Changes after THA were reported for groups 1 and 2 representing spinopelvic complex key parameter lumbar flexibility (∆ LL), pelvic mobility (∆ SS) and hip motion (∆ PFA), but not for group 3. This is the first study to present age-adjusted normative values for spinopelvic mobility. The subgroups with increased age were identified as risk cohort for altered spinopelvic mechanics and enhanced sagittal spinal imbalance and limited capacity for improvement of mobility after THA. This valuable information serves to focus in the preoperative screening on the THA candidates with the highest risk for abnormal spinopelvic function.

## Introduction

The spinopelvic complex recently gained attention in the etiology of hip arthroplasty instability^1–6^. It represents the kinetic chain with mutual interactions between the hip, pelvis and spine providing adaption processes during changes of position and therefore enabling erect posture and horizontal gaze^7–9^.

The spinopelvic mobility is represented by the changes in everyday movements such as changing posture from standing to sitting in the main spinopelvic elements lumbar flexibility (∆ LL = LL_standing_ − LL_sitting_), pelvic mobility (∆ SS = _standing_ − SS_sitting_) and hip motion (∆ PFA = PFA_standing_ − PFA_sitting_). Pelvic mobility is further defined as stiff (∆ SS < 10°), normal (∆ SS ≥ 10°–30°), and hypermobile (∆ SS > 30°)^10^.

Degenerative spine and hip pathologies may alter the spinopelvic mechanics leading to abnormal spinopelvic mobility^10–14^. Functional limitations in certain segments of the spinopelvic complex are compensated for by the remaining segments, which is expressed in the newly defined “hip user index”, in which restricted lumbar flexibility (∆ LL), is compensated for by increased hip motion (∆ PFA)^15^. It is known, that patients with less lumbar flexibility (∆ LL), reduced pelvic mobility (∆ SS) and enhanced hip motion (∆ PFA) have a significantly increased risk of total hip arthroplasty (THA) dislocation and an inferior outcome^14,16–19^. Not only restricted spinopelvic mobility, but also hypermobility is associated with poorer outcome and enhanced THA instability^20^.

Consequently, to mitigate the risk of THA dislocations, spinopelvic mobility and to a smaller extent sagittal spinal balance recently received some attention from arthroplasty surgeons in terms of acetabular component positioning adapted to the individual spinopelvic mobility^10,21–23^.

However, the real challenge is to identify THA patients in the preoperative screening, who really benefit from an additional complex diagnostic procedure associated with a greater logistical and financial burden and radiation exposure. For this purpose, it is necessary to know which patients have abnormal spinopelvic mobility. In this context, it is still unknown how the spinopelvic complex is influenced by age and how the abnormal spinopelvic mobility is changed by age groups.

The aim of our study is therefore (1) to determine the association of age on the individual segments of the spinopelvic complex and spinal sagittal alignment in patients undergoing hip replacement and (2) to assess how the pre- and postoperative pelvic mobility changes depending on the age groups after THA. This is the first time that pre- and postoperative normative values for spinopelvic mobility are defined for different age groups in THA patients.

## Materials and methods

A prospective radiological observational study on patients undergoing primary total hip replacement in an orthopedic university hospital between September 2019 and November 2020 was performed. The study is in compliance with the Helsinki Declaration, has been approved by the institutional ethics board (EA2/142/17) and patients have given their informed written consent. The exclusion criteria were defined as non-elective surgery, bilateral planned THA, severe hip dysplasia with subsequent THA and femur osteotomy, any form of revision THA, ankylosing spondylitis, spinal fusion surgery at any level, osseous metastasis and neurological pre-existing conditions that significantly influence posture. The prosthesis components and fixation techniques (Supplement Table 1) were selected according to the patient's individual requirements and planned preoperatively using TraumaCad (Brainlab, Munich, Germany). The indications for THA of the included patients were primary osteoarthritis of the hip (n = 144), secondary osteoarthritis of the hip divided into the following subgroups adult hip dysplasia (n = 21), avascular necrosis of the head (n = 14), femoroacetabular impingement type CAM (n = 9), others (n = 9).

### Radiographic assessment and measurement protocols

**Figure 1:**
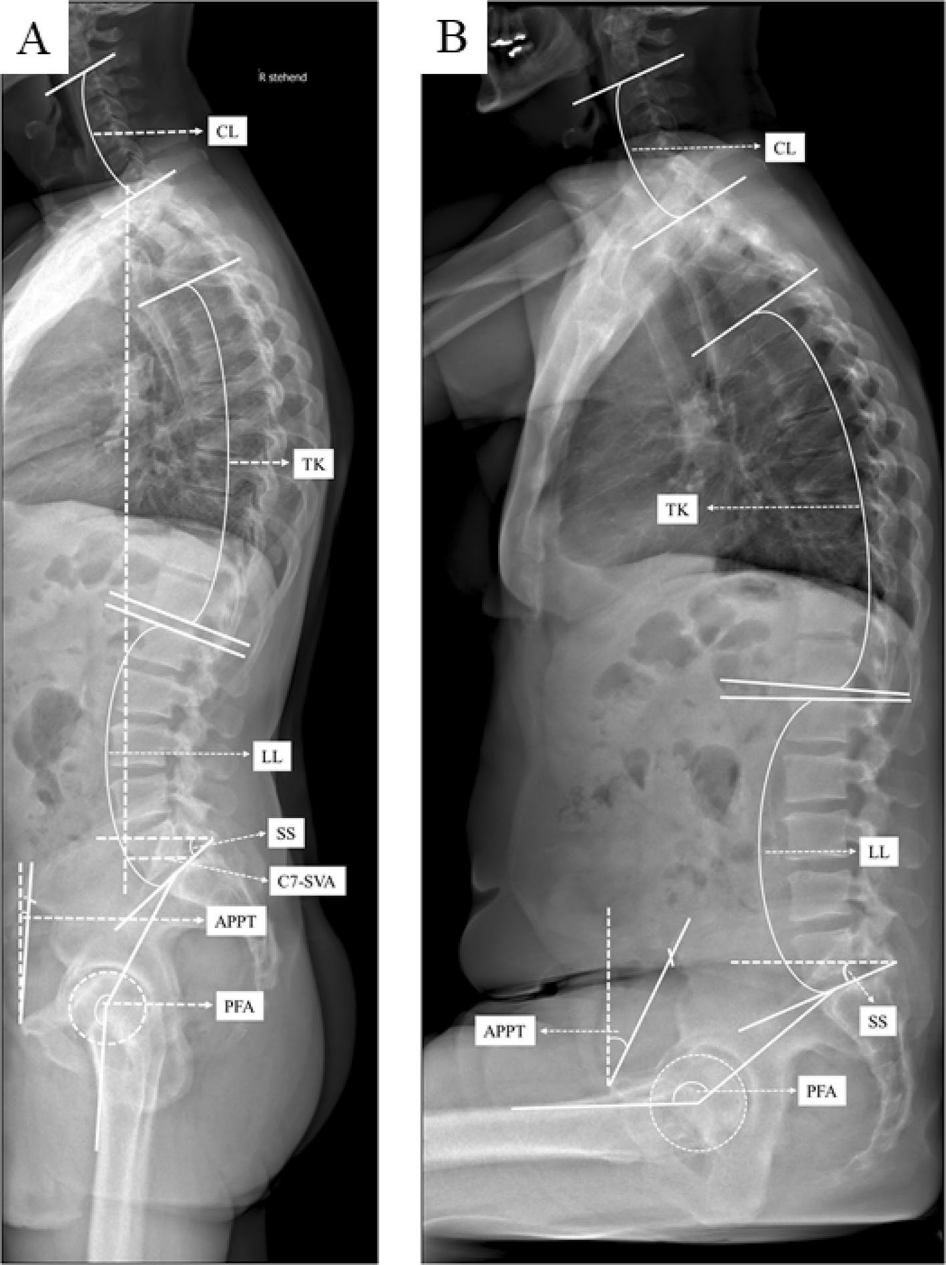
Sagittal standing (**A**) and sitting (**B**) EOS radiographs of the spine, pelvis and hip depicting global spinal balance C7-sagittal vertical axis (C7-SVA), sagittal spinal parameter cervical lordosis (CL), thoracic kyphosis (TK) and spinopelvic parameter lumbar lordosis (LL), sacral slope (SS), pelvic incidence (PI), anterior plane pelvic tilt (APPT) and pelvic femoral angle (PFA).

The radiographic assessments were conducted by an experienced orthopedic surgeon using Merlin Diagnostic Workcenter (Phoenix PACS, Freiburg, Germany) and a randomly selected 25% dataset was measured by a second independent orthopedic surgeon^24^. Following parameter have been measured pre- and postoperatively (Fig. 1, Supplement Table 2 for definition): C7-Sagittal vertical axis (C7-SVA; balance ≤ 50 mm; imbalance > 50 mm), cervical lordosis (CL), thoracic kyphosis (TK), lumbar lordosis (LL), pelvic incidence (PI), anterior plane pelvic tilt (APPT), sacral slope (SS), pelvic femoral angle (PFA). Key parameters of the spinopelvic complex are defined as lumbar flexibility (∆ LL = LL_standing_ − LL_sitting_), pelvic mobility (∆ SS = _standing_ − SS_sitting_) and hip motion (∆ PFA = PFA_standing_ − PFA_sitting_). Pelvic mobility is further defined based on ∆ SS = SS_standing_ − SS_sitting_ as stiff (∆ SS < 10°), normal (∆ SS ≥ 10°–30°), and hypermobile (∆ SS > 30°)^10^. The patient collective was classified into group 1 < 60 years, group 2 ≥ 60–79 years and group 3 ≥ 80 years. Within 3 days pre- and five to seven days postoperatively, each patient got a complete spine imaging including the pelvis up to the proximal tibia from lateral and anterior–posterior in standing position using biplanar low dose stereoradiography (EOS, Paris, France). Patients are advised to stand naturally, look forward and place their hands on a support with relaxed upper limbs and were instructed to sit relaxed in the seated position on a height-adjustable stool with the femur parallel to the floor.

### Statistical analyses

All statistical analyses were performed using SPSS Version 27 (IBM Corporation, New York, United States). We used the paired t-test for the pre- to postoperative comparison. Analysis of variance (ANOVA) and post-hoc analysis according to Games-Howell was used to determine differences between the age groups according to the spinopelvic and spinal parameters. Spearman’s rank correlation coefficient was used to determine the interrater reliability of the radiographic measurements. Differences between the ratios of spinopelvic mobility classification and the age groups preoperatively and postoperatively were analyzed using Fisher’s exact test due to the small number of cases (< 5) in some groups. A statistical power analysis (G*power 3.1.9.7) was performed for post hoc power estimation based on preoperative and postoperative data (sample size, means, standard deviation and estimated effect size) from our study^25^. We performed two power analysis one for the comparison between the age groups (ANOVA) and one for the comparison from preoperative to postoperative regarding pelvic mobility stratified for age groups. A significance level of p < 0.05 was assumed for all tests.

### Institutional review board

The study has been approved by the institutional ethics board of Charité—Universitätsmedizin Berlin and is registered as approved study with the number EA2/142/17.

## Results

**Figure 2:**
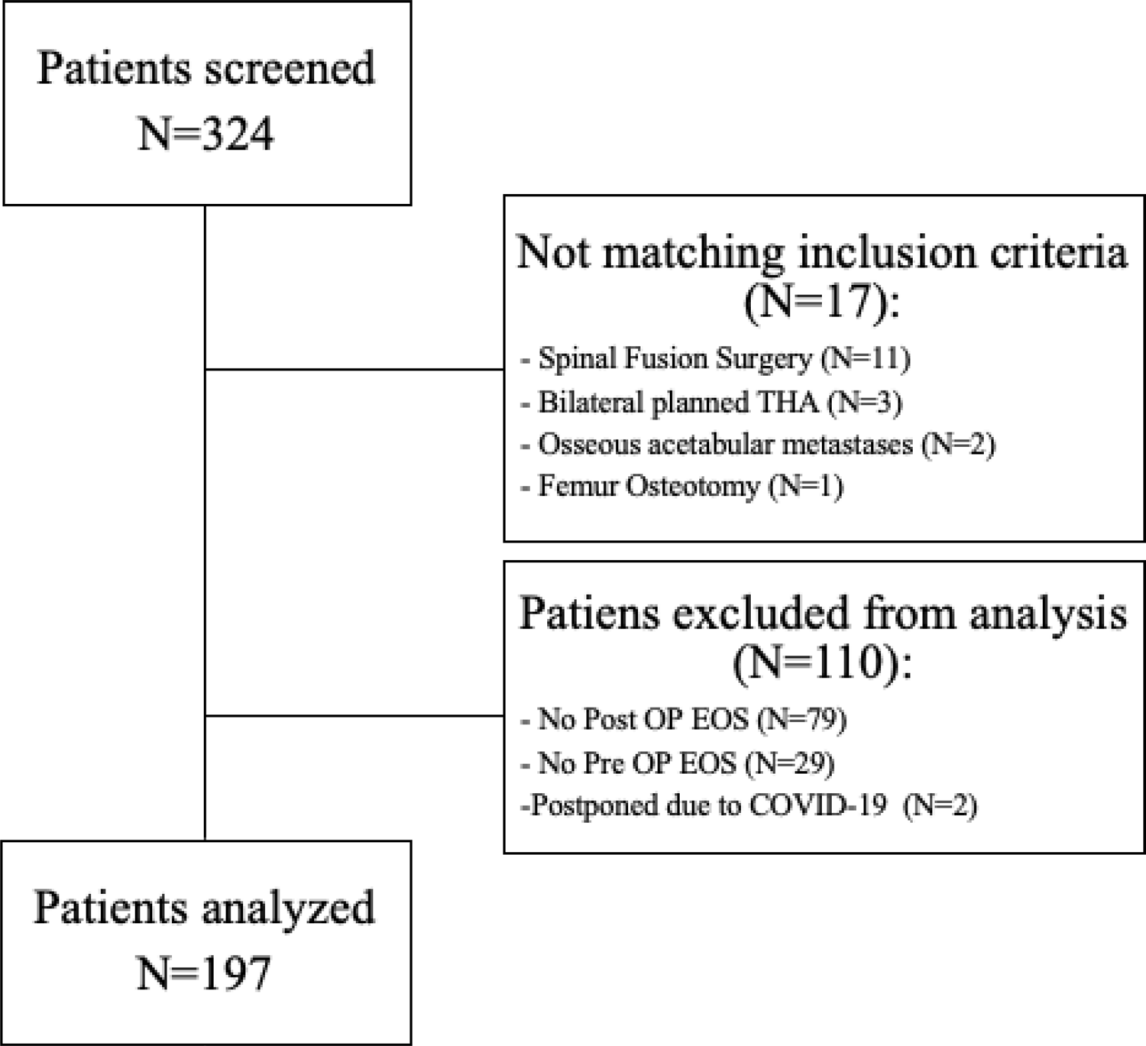
Study flow of patient inclusion and exclusion.

A total of 324 primary THA patients were screened for study eligibility, 197 patients met the inclusion and no exclusion criteria. (Fig. 2) There were 106 female and 91 male patients with a mean age of 66.3 years (range 17–88 years) and a mean BMI of 26.8 kg/m^2^. Interrater reliability analysis demonstrated good interobserver agreements (Supplement Table 3)^26^. We classified the patient collective into three groups: group 1 < 60 years (n = 56), group 2 ≥ 60–79 years (n = 112) and group 3 ≥ 80 years (n = 29).

### Age and spinopelvic function

Spinopelvic key parameter lumbar flexibility (∆ LL) was decreased with increasing age preoperatively (groups 1/2/3: 28.7°/20.0°/16.3°) (Table 1) and postoperatively (groups 1/2/3: 36.1°/23.1°/17.2°) (Table 2, Supplemental Fig. 1). The other key element for spinopelvic function pelvic mobility (∆ SS = SS_standing_ − SS_sitting_) was the highest in the youngest aged patients (groups 1/2/3: 19.3°/17.3°/16.8°) preoperatively. Postoperative pelvic mobility revealed enhancements above all age groups after THA with increases of the group < 60 years compared to both older aged groups (groups 1/2/3: 27.8°/21.0°/17.9°) (Supplemental Fig. 2). Pelvic retroversion (APPT standing: + values indicating anterior pelvic posture and − values posterior pelvic posture in the sagittal plane) increased with increasing age preoperatively (groups 1/2/3: 3.7°/− 0.6°/− 0.4°) and postoperatively (groups 1/2/3: 6.9°/1.9°/− 0.5°). These results demonstrated less pelvic retroversion postoperatively and an increased change after hip replacement with decreasing age.

**Table 1:** Analysis of spinopelvic complex elements lumbar flexibility (∆ LL = LL_standing_ − LL_sitting_), pelvic mobility (∆ SS = SS_standing_ − SS_sitting_) respectively change in pelvic tilt (∆ APPT = APPT_standing_ − APPT_sitting_ and hip motion (∆ PFA = PFA_standing_ − PFA_sitting_) and spinopelvic parameter LL, APPT, SS, PFA and PI in standing position according to the age: group 1 < 60 years, group 2 ≥ 60–79 years and group 3 ≥ 80 years preoperatively.

Preoperative spinopelvic parameter according to the age						
Age groups	< 60 years Preoperative mean (± SD)	≥ 60–79 years Preoperative mean (± SD)	≥ 80 years Preoperative mean (± SD)	p-value 95%CI (#1)	p-value 95%CI (#2)	p-value 95%CI (#3)
∆ LL [°]	28.7 (13.0)	20.0 (10.7)	16.3 (10.9)	0.000 [4.0; 13.5]	0.251 [− 1.9; 9.3]	0.000 [6.0; 18.9]
∆ APPT [°]	22.9 (11.2)	18.7 (9.0)	18.5 (7.5)	0.054 [− 0.1; 8.3]	0.984 [− 6.2; 5.4]	0.096 [− 6.3; 6.1]
∆ SS [°]	19.3 (11.2)	17.3 (9.5)	16.8 (11.3)	0.482 [− 2.1; 6.2]	0.972 [− 5.1; 6.2]	0.597 [− 3.8; 8.8]
∆ PFA [°]	57.3 (15.3)	55.9 (15.3)	56.5 (17.0)	0.851 [− 4.6; 7.4]	0.983 [− 9.2;8.0]	0.979 [− 8.5; 10.0]
LL Stand [°]	55.2 (11.6)	50.3 (15.4)	48.1 (13.3)	0.061 [− 0.2; 9.9]	0.721 [− 4.7;9.2]	0.049 [0; 14.1]
APPT Stand [°]	3.7 (6.3)	− 0.6 (8.1)	− 0.4 (9.7)	0.001 [1.6; 7.0]	0.994 [− 5.0;4.6]	0.112 [− 0.7; 8.9]
SS Stand [°]	42.6 (9.1)	40.2 (10.6)	41.0 (12.8)	0.271 [− 1.3; 6.2]	0.942 [− 7.1;5.4]	0.820 [− 4.9; 8.1]
PFA Stand [°]	179.4 (12.2)	178.8 (10.7)	181.3 (10.4)	0.947 [− 4.0; 5.2]	0.466 [− 7.9;2.7]	0.713 [− 8.0; 4.1]
PI Stand [°]	54.4 (11.0)	54.4 (12.6)	57.5 (15.9)	1.0 [− 4.5; 4.5]	0.603 [− 10.8; 4.7]	0.629 [− 11.1; 5.0]

p-values indicating differences between groups < 60 years and ≥ 60–79 years (#1), ≥ 60–79 years and ≥ 80 years (#2) and < 60 years and ≥ 80 years (#3). ANOVA and post-hoc analysis according to Games-Howell were used and level of significance set at p < 0.05.

*SD* standard deviation, *95%CI* 95% confidence interval.

**Table 2:** Analysis of spinopelvic complex elements lumbar flexibility (∆ LL = LL_standing_ − LL_sitting_), pelvic mobility (∆ SS = SS_standing_ − SS_sitting_) respectively change in pelvic tilt (∆ APPT = APPT_standing_ − APPT_sitting_ and hip motion (∆ PFA = PFA_standing_ − PFA_sitting_) and spinopelvic parameter LL, APPT, SS, PFA and PI in standing position according to the age: group 1 < 60 years, group 2 ≥ 60–79 years and group 3 ≥ 80 years postoperatively.

Postoperative spinopelvic parameter according to the age						
Age groups	< 60 years Postoperative mean (± SD)	≥ 60–79 years Postoperative mean (± SD)	≥ 80 years Postoperative mean (± SD)	p-value 95%CI (#1)	p-value 95%CI (#2)	p-value 95%CI (#3)
∆ LL [°]	36.1 (11.1)	23.1 (9.9)	17.2 (10.1)	0.000 [8.8; 17.2]	0.020 [0.8; 11.1]	0.000 [13.1; 24.7]
∆ APPT [°]	27.3 (11.5)	22.6 (9.5)	20.1 (9.9)	0.027 [0.4; 9.0]	0.458 [− 2.6; 7.6]	0.012 [1.4; 13.0]
∆ SS [°]	27.8 (9.7)	21.0 (9.5)	17.9 (11.2)	0.000 [3.0; 10.6]	0.371 [− 2.4; 8.6]	0.001 [4.0; 15.8]
∆ PFA [°]	46.1 (14.2)	51.7 (12.5)	52.9 (14.5)	0.037 [− 10.9; − 0.3]	0.904 [− 8.4; 5.9]	0.102 [− 14.8; 1.1]
LL Stand [°]	55.9 (11.3)	51.2 (14.5)	50.0 (14.4)	0.060 [− 0.2; 9.5]	0.914 [− 6.1; 8.5]	0.145 [− 1.6; 13.3]
APPT Stand [°]	6.9 (6.7)	1.9 (7.4)	− 0.5 (8.1)	0.000 [2.3; 7.6]	0.330 [− 1.6; 6.4]	0.000 [3.2; 11.7]
SS Stand [°]	45.1 (8.1)	42.1 (10.3)	42.2 (11.7)	0.109 [− 0.5; 6.4]	1.0 [− 5.8; 5.7]	0.458 [− 3.0; 8.8]
PFA Stand [°]	172.9 (13.0)	176.2 (9.7)	176.7 (9.3)	0.206 [− 8.0; 1.3]	0.972 [− 5.2; 4.3]	0.273 [− 9.7; 2.1]
PI Stand [°]	52.3 (10.9)	53.9 (12.7)	56.6 (15.3)	0.679 [− 6.1; 2.9]	0.661 [− 10.2; 4.8]	0.381 [− 12.0; 3.5]

p-values indicating differences between groups < 60 years and ≥ 60–79 years (#1), ≥ 60–79 years and ≥ 80 years (#2) and < 60 years and ≥ 80 years (#3). ANOVA and post-hoc analysis according to Games–Howell were used and level of significance set at p < 0.05.

*SD* standard deviation, *95%CI* 95% confidence interval.

### Age and sagittal spinal alignment

Sagittal alignment based on C7-SVA demonstrated greater imbalance in age groups ≥ 60–79 years and ≥ 80 years compared to the < 60 years group preoperatively (61.5 mm and 71.5 mm vs. 28.6 mm) and postoperatively (60.4 mm and 71.2 mm vs. 34.5 mm) (Table 3). In summary, there is a distinct association between increasing age and greater sagittal imbalance. Remarkably was the severity of imbalance in both older age groups of our THA collective, following the widely accepted definition of sagittal imbalance of > 50 mm in the C7-SVA^27^.

**Table 3:** Analysis of global sagittal alignment parameter C7-central vertical axis and spinal sagittal parameter in standing position and during motion, cervical lordosis (CL) and ∆ CL = CL_standing_ − CL_sitting_ and thoracic kyphosis (TK) and ∆ TK = TK_standing_ − TK_sitting_ according to the age: group 1 < 60 years, group 2 ≥ 60–79 years and group 3 ≥ 80 years.

Sagittal spinal alignment according to the age							
Age groups		< 60 years Mean (± SD)	≥ 60–79 years mean (± SD)	≥ 80 years Mean (± SD)	p-value (#1)	p-value (#2)	p-value (#3)
C7-SVA [mm]	Pre	28.6 (25.3)	61.5 (40.0)	71.5 (39.0)	0.000	0.451	0.000
	Post	34.5 (27.9)	60.4 (32.3)	71.2 (39.7)	0.000	0.376	0.000
CL stand [°]	Pre	14.2 (10.6)	16.6 (11.8)	16.4 (10.4)	0.413	0.996	0.649
	Post	12.7 (11.6)	16.1 (10.9)	15.4 (9.1)	0.176	0.930	0.496
∆ CL [°]	Pre	3.0 (6.9)	1.9 (7.4)	3.9 (5.4)	0.608	0.269	0.819
	Post	3.7 (7.2)	1.7 (7.5)	1.5 (6.5)	0.231	0.983	0.335
TK stand [°]	Pre	38.7 (10.1)	42.9 (12.5)	38.9 (9.2)	0.052	0.152	0.990
	Post	36.3 (10.9)	40.7 (12.0)	37.9 (9.0)	0.054	0.367	0.763
∆ TK [°]	Pre	4.0 (6.1)	0.6 (5.8)	1.7 (5.1)	0.002	0.118	0.000
	Post	3.6 (8.9)	1.4 (5.5)	0.1 (5.2)	0.205	0.377	0.046

p-values indicating differences between groups < 60 years and ≥ 60–79 years (#1), ≥ 60–79 years and ≥ 80 years (#2) and < 60 years and ≥ 80 years (#3). ANOVA and post-hoc analysis according to Games-Howell were used and level of significance set at p < 0.05.

*SD* standard deviation, *Pre* preoperatively, *Post* postoperatively.

### Age and mobility before and after hip arthroplasty

**Figure 3:**
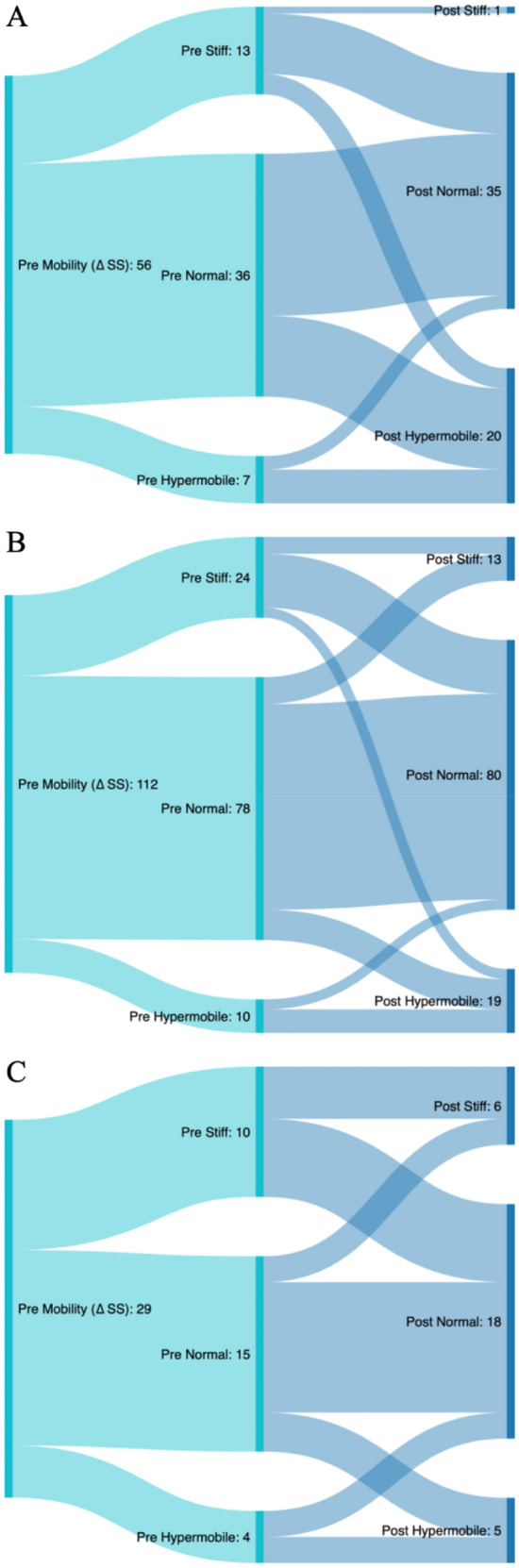
Sankey analysis depicting the flow from preoperative to postoperative pelvic mobility in each age group based on ∆ SS = SS_standing_-SS_sitting_ defined as stiff (∆ SS < 10°), normal (∆ SS ≥ 10°-30°), and hypermobile (∆ SS > 30°). (**A**) < 60 years, (**B**) group 2 ≥ 60–79 years and group 3 ≥ 80 years. *Pre* preoperative, *Post* postoperative, *SS* sacral slope.

The preoperative proportion of stiff patients in terms of pelvic mobility in group < 60 years is distinctly lower than in group ≥ 80 years (23.2% vs. 35.7%). Hip replacement decreased the number of patients categorized with stiff pelvic mobility in all age groups. The largest reduction was demonstrated in the group aged < 60 years. Reciprocally, the proportion of patients classified with hypermobile mobility was increased in all age groups after THA, with the proportion of < 60 years group rising the most (preoperatively 12.5% to postoperatively 35.7%). The same pattern was seen for lumbar flexibility, which was greater preoperatively in group < 60 years than in group ≥ 80 years (28.7° vs 16.3°) and increased more postoperatively in group 1 than in group 3 (36.1° vs 17.2°) (Tables 1, 2). In summary, with increasing age, the proportion of preoperative pelvic and lumbar stiffness increased and the postoperative alterations after THA decreased (Table 4, Supplement Table 5, Fig. 3, Supplemental Fig. 3).

**Table 4:** Contribution of pre- and postoperative pelvic mobility based on ∆ SS = SS_standing_ − SS_sitting_ defined as stiff (∆ SS < 10°), normal (∆ SS ≥ 10°–30°), and hypermobile (∆ SS > 30°) according to the age: group 1 < 60 years, group 2 ≥ 60–79 years and group 3 ≥ 80 years.

Classification of pre-and postoperative pelvic mobility according to the age				
Pelvic Mobility (∆ SS)		< 60 years	≥ 60–79 years	≥ 80 years
Stiff [%/N]	Pre	23.2 (13)	21.4 (24)	34.5 (10)
	Post	1.8 (1)	11.6 (13)	20.7 (6)
Normal [%/N]	Pre	64.3 (36)	69.6 (78)	51.7 (15)
	Post	62.5 (35)	72.3 (81)	62.1 (18)
Hypermobile [%/N]	Pre	12.5 (7)	8.9 (10)	13.8 (4)
	Post	35.7 (20)	16.1 (18)	17.2 (5)

% represents the percentage contribution.

*N* absolute number of patients, *pre* preoperative, *post* postoperative.

### Pre to postoperative changes in spinopelvic and spinal parameter according to the age

Changes after THA were reported for groups 1 and 2 representing spinopelvic complex key parameter lumbar flexibility (∆ LL), pelvic mobility (∆ SS and ∆ APPT) and hip motion (∆ PFA), but not for group 3 (≥ 80 years), which is expressed in the low ability of patients with increased age to improve mobility after THA (Table 5).

**Table 5:** Differences between pre- and postoperative spinopelvic and spinal sagittal parameter a according to the age: group 1 < 60 years, group 2 ≥ 60–79 years and group 3 ≥ 80 years postoperatively.

Differences between pre- and postoperative spinopelvic parameter according to the age									
Parameter	< 60 years			≥ 60–79 years			≥ 80 years		
	Pre	Post	p-value	Pre	Post	p-value	Pre	Post	p-value
∆ CL	3.02 (6.9)	3.72 (7.2)	0.532	1.92 (7.4)	1.74 (7.5)	0.832	3.86 (5.4)	1.49 (6.5)	0.105
∆ TK	4.03 (6.1)	3.61 (8.9)	0.760	0.57 (5.8)	1.36 (5.5)	0.245	1.66 (5.1)	0.12 (5.2)	0.311
∆ LL	28.71 (13.0)	36.09 (11.1)	0.000	19.98 (10.7)	23.12 (9.9)	0.001	16.27 (10.9)	17.19 (10.2)	0.861
∆ APPT	22.87 (11.2)	27.29 (11.5)	0.001	18.73 (9.0)	22.6 (9.5)	0.000	18.45 (7.5)	20.08 (9.9)	0.976
∆ PFA	57.27 (15.3)	46.06 (14.2)	0.000	55.9 (15.3)	51.67 (12.5)	0.006	56.52 (17.0)	52. 9 (14.5)	0.363
∆ SS	19.32 (11.2)	27.79 (9.7)	0.000	17.30 (9.5)	21.0 (9.5)	0.000	16.78 (11.3)	17.9 (11.2)	0.963
CL_Stand_	14.25 (10.6)	12.74 (11.6)	0.127	16.55 (11.8)	16.10 (10.9)	0.519	16.37 (10.4)	15.37 (9.1)	0.566
TK_Stand_	38.65 (10.1)	36.33 (10.9)	0.004	42.87 (12.5)	40.65 (12.0)	0.000	38.94 (9.2)	37.89 (9.0)	0.302
LL_Stand_	55.17 (11.6)	55.91 (11.2)	0.365	50.31 (15.4)	51.23 (14.5)	0.071	48.09 (13.3)	50.01 (14.4)	0.043
APPT_Stand_	3.66 (6.3)	6.91 (6.7)	0.000	0.63 (8.1)	1.86 (7.4)	0.000	0.43 (9.7)	0.53 (8.1)	0.940
PFA_Stand_	179.36 (12.2)	172.87 (12.0)	0.000	178.76 (10.7)	176.23 (9.7)	0.005	181.34 (10.4)	176.67 (9.3)	0.023
SS_Stand_	42.61 (9.1)	45.10 (27.8)	0.001	40.17 (10.6)	42.13 (10.3)	0.001	41.01 (12.8)	42.18 (11.7)	0.283

p-values indicating differences between pre- and postoperative values. Paired t-test was used, and level of significance set at p < 0.05.

## Discussion

The investigation of 197 prospectively enrolled patients undergoing primary hip replacement with pre- and post-operative EOS assessment in standardized standing and relaxed sitting position demonstrated significant decreases of lumbar flexibility and pelvic mobility and increases of hip motion and standing pelvic retroversion with increasing age. Global spinal sagittal imbalance revealed higher in the age groups ≥ 60–79 years and ≥ 80 years compared to < 60 years. There was a distinct disparity in the distribution of patients in terms of pelvic mobility with a larger proportion of spinopelvic stiffness and a smaller proportion of hypermobility in the group ≥ 80 years compared to the group < 60 years.

In recent years, pathological spinopelvic mobility has received considerable attention as a risk factor for THA dislocations. Additional complex diagnostics with radiographs of the spine, the pelvis and femur in different positions from standing to relaxed sitting even to deep flexed sitting was recommended in the preoperative diagnostics to identify patients at risk and to adjust the implant position accordingly^10,15,21,22,28^. While instrumented spinal fusion is a known risk factor for THA dislocation, one study reported that the majority of THA candidates with limited lumbar range of motion do not have spinal fusion and degenerative causes are responsible for the limitation^29^. They, therefore, recommended that a spinopelvic assessment with functional radiographs need to be performed preoperatively in all THA candidates^30^. From our point of view, the patient groups with the highest prevalence of altered spinopelvic mechanics need to be identified for functional assessment to minimize radiation exposure, logistical and financial effort. This fundamental analysis helps surgeons to identify these subgroups of patients. The distinct relationship between age and spinopelvic function was reflected in the reduced pelvic mobility of the older patient groups ≥ 60–79 years and ≥ 80 years preoperatively and postoperatively. This may lead to a lack of acetabular opening during sitting with anterior impingement and posterior dislocation and is an established risk factor for THA instability^2,13^.

The same pattern was demonstrated in the reduced lumbar flexibility of the older patient groups ≥ 60–79 years and ≥ 80 years preoperatively and postoperatively. Other investigations have classified lumbar flexibility of less than 20° as spinal stiffness. In our study, this severe restriction was seen pre- and postoperatively in the age group ≥ 80 years. Restricted lumbar flexibility is a known risk factor for THA dislocations, in relation to spinal fusion, but also to degenerative alterations^13,28,29^. The restricted lumbar flexibility was considered to be compensated by increased hip motion. This compensation mechanism was also reflected in our data, with increased ∆ PFA in the older age groups known for limited pelvic and lumbar mobility. This mechanism of femoral compensation has recently been defined in the “hip user index” and is a suspected driving mechanism leading to fermoracetabular impingement and subsequent THA dislocation^15^. Both, limited lumbar flexibility and increased hip motion are therefore considered risk factors for THA instability^13,14^.

In addition to the age-dependent spinopelvic functional impairment, there were also other age-dependent changes. The increased global sagittal imbalance in the older patients (≥ 60–79 years and ≥ 80 years) was particularly striking. Sagittal spinal imbalance was also discussed as a contributing factor to THA instability^28,31^. This was explained by the interaction of the pelvis and vertebral column in facilitating upright posture^32–34^. Sagital spinal imbalance leaded to a pelvic compensation mechanism with increased pelvic retroversion, which is already described in spine literature^35–39^. This phenomenon was clearly visible in our data, patients with increasing age and increased sagittal imbalance, demonstrated significantly greater pelvic retroversion. While it should be noted, that increased pelvic retroversion in standing was reported as an associated factor for unfavourable pelvic mobility and affects the functional acetabular orientation with increased acetabular cup inclination and anteversion^1,40^. Accordingly sagittal spinal malalignment in THA patients has been related to a high prevalence of excessively anteverted acetabular components^36^. As a result an investigation demonstrated an increased risk of dislocation in THA patients with spinal sagittal imbalance^35^.

The described pelvic compensation mechanism was reported to lead to alterations in sacroiliac joints due to biomechanical strains as a result PI increased with concomitant increasing age in sagittal imbalanced patients^41^. In our investigation a similar pathomechanism might be considered, while a larger PI in older age groups was demonstrated. When highlighting the contribution of PI, there is evidence that considered high PI as a risk indicator for THA instability^42^.

The varying distribution of pelvic mobility in terms of the age groups with distinctly more stiffness in patients’ ≥ 80 years demonstrated the need for an age adjusted assessment of spinopelvic function. There were 35.7% of the patients’ ≥ 80 years with limited pelvic mobility preoperatively, which was in line with the reported prevalence of 34.2% stiff pelvic mobility in a retrospective investigation of THA candidates. Nevertheless this study investigated a patient cohort with a mean age of 60 years, the similar proportion of stiffness in these younger patient cohort was might be explained by the inclusion of patients with spinal fusion surgery and subsequent restricted spinopelvic mobility^23^.

In addition, the postoperative changes after THA in patients < 60 years need to be emphasized. The proportion of pelvic stiffness decreased markedly in the group (< 60 years) from 20.7% to only 1.8% postoperatively. The distinctly greater resources of improvement after THA in the younger patients cohort (< 60 years) was also evident in lumbar flexibility, which increased from 28.7° to 36.1°, compared with only 16.3° to 17.2° in the older patients (≥ 80 years). It was assumed that the restricted pelvic mobility in the younger patients was caused by the osteoarthritis of the hip and could be effectively improved by the THA. Whereas the older patients with a presumably longer course of osteoarthritis, on the one hand, develop more additional contractures, but are also generally exposed to advancing degenerative changes in other segements of the spinopelvic complex. Thus, the possibility of improvement through THA only is limited in the group ≥ 80 years. Attention should also be paid to the postoperative increase in the proportion of pelvic hypermobility in the younger patients cohort (< 60 years) from 12.5 to 35.7%. As it is known that hypermobility in combination with spinal fusion in THA patients leads to an inferior outcome and more instability^20^. The poor postoperative improvement in spinopelvic function was reflected in the lack of significant postoperative alterations in lumbar flexibility, pelvic mobility and hip motion in patients ≥ 80 years.

Some limitations of the study need to be addressed. EOS assessments were performed during hospitalization and only short-term follow-up was presented, but long-term follow-up is planned. It should be critically noted that the examination is in the immediate postoperative phase on which factors such as pain may have had an influence on the physiological spinopelvic function. To minimize the effect of pain on spinopelvic function, we monitored our THA patients as standard and individually adjusted their pain medication according to a standardized pain management protocol, which was developed in cooperation with the pain service of the anesthesiology department. In our study, the relaxed seated position was selected as the functional assessment and a deep flexed seated or single-leg standing position was not performed as an additional functional exercise. The deep flexed seated position might be an advantage when identifying “hip users”. These functional images were not possible in the postoperative setting due to patient safety^5,15,43^. When interpreting our results as age-adjusted normative values for spinopelvic mobility, a selection bias due to the European population and the possibly non-representative selection of study patients, e.g. exclusion of spinal fusion surgery, should be considered. Furthermore, a point to consider is that part of the study was conducted during the SARS-CoV-2 pandemic. The pandemic had a drastic impact on health care systems and surgical capabilities. Therefore, a selection bias for the patient selection cannot be ruled out, which reduces the generalizability of our results. Another limitation of the study is that no control for multiplicity was performed. This must be critically considered when interpreting the results.

The study is the first to present age-adjusted normative values for spinopelvic mobility, and identified the subgroups with increased age (≥ 60–79 years and ≥ 80 years) as risk cohort for altered spinopelvic mechanics. This assessment showed restrictions in lumbar flexibility and pelvic mobility, more sagittal spinal imbalance, and a lower postoperative possibility of improvement with increasing age. This investigation clearly demonstrated the importance of age-adjusted consideration of spinopelvic mobility with a particular focus in preoperative spinopelvic assessment of THA candidates on elderly patients.

## Supplementary Information

Supplementary Information.

## Data Availability

The datasets generated and analyzed during the current study are available from the corresponding author on reasonable request.
