# Supplementary material for: Association of age and spinopelvic function in patients receiving a total hip arthroplasty
Source: Sci Rep. 2023 Feb 14;13:2589. doi: 10.1038/s41598-023-29545-5 (PMC9929091; doi:10.1038/s41598-023-29545-5)
Supplement: Supplementary file 1 — Supplementary Information. [file 41598_2023_29545_MOESM1_ESM.docx]

# Supplements


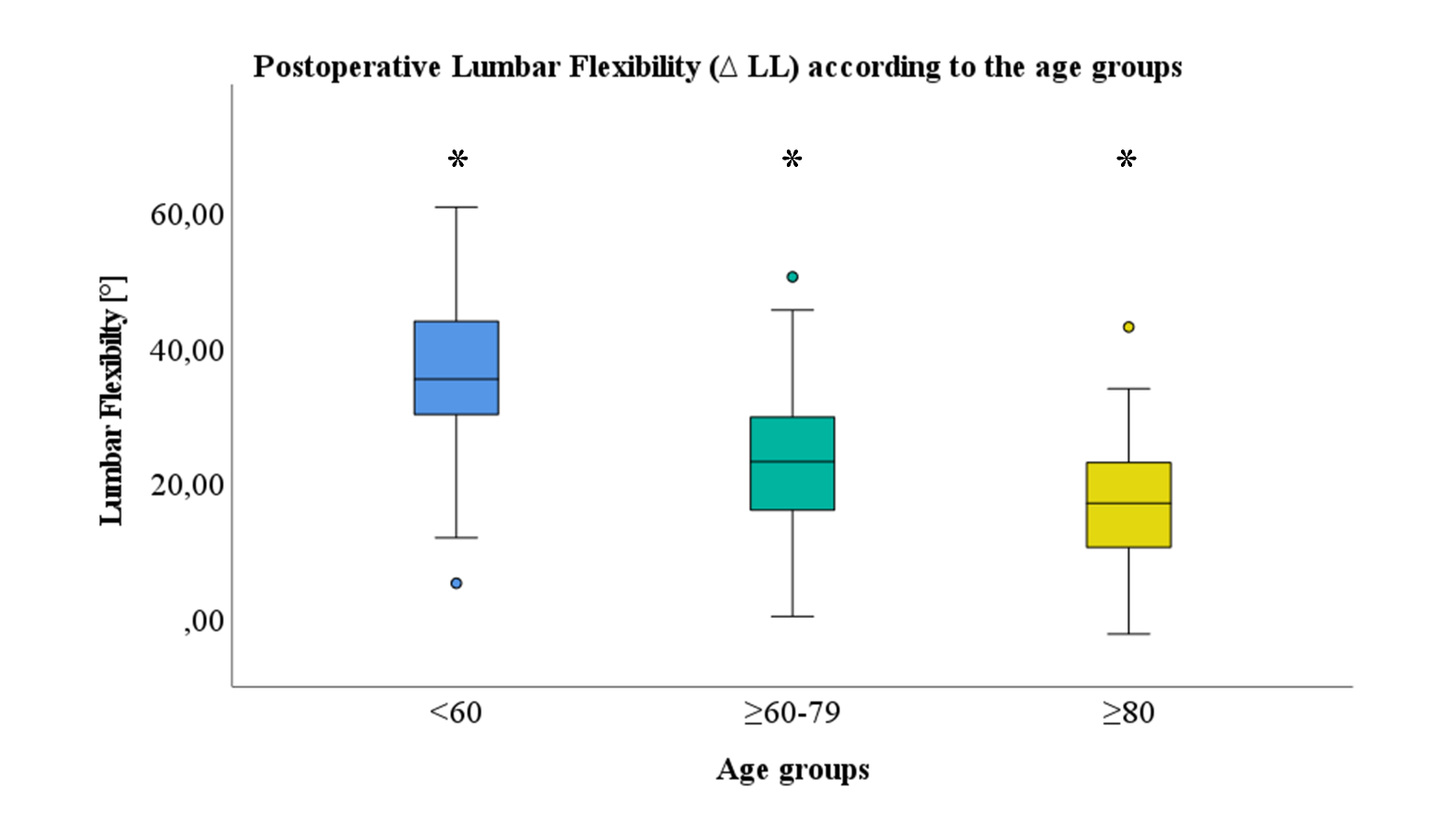


**Supplement figure 1.** Postoperative lumbar flexibility (∆LL) as a key segment of the spinopelvic complex is depicted in relation to the defined age: group 1 < 60 years, group 2 ≥60-79 years and group 3 ≥80 years. * indicating significant differences between all groups.


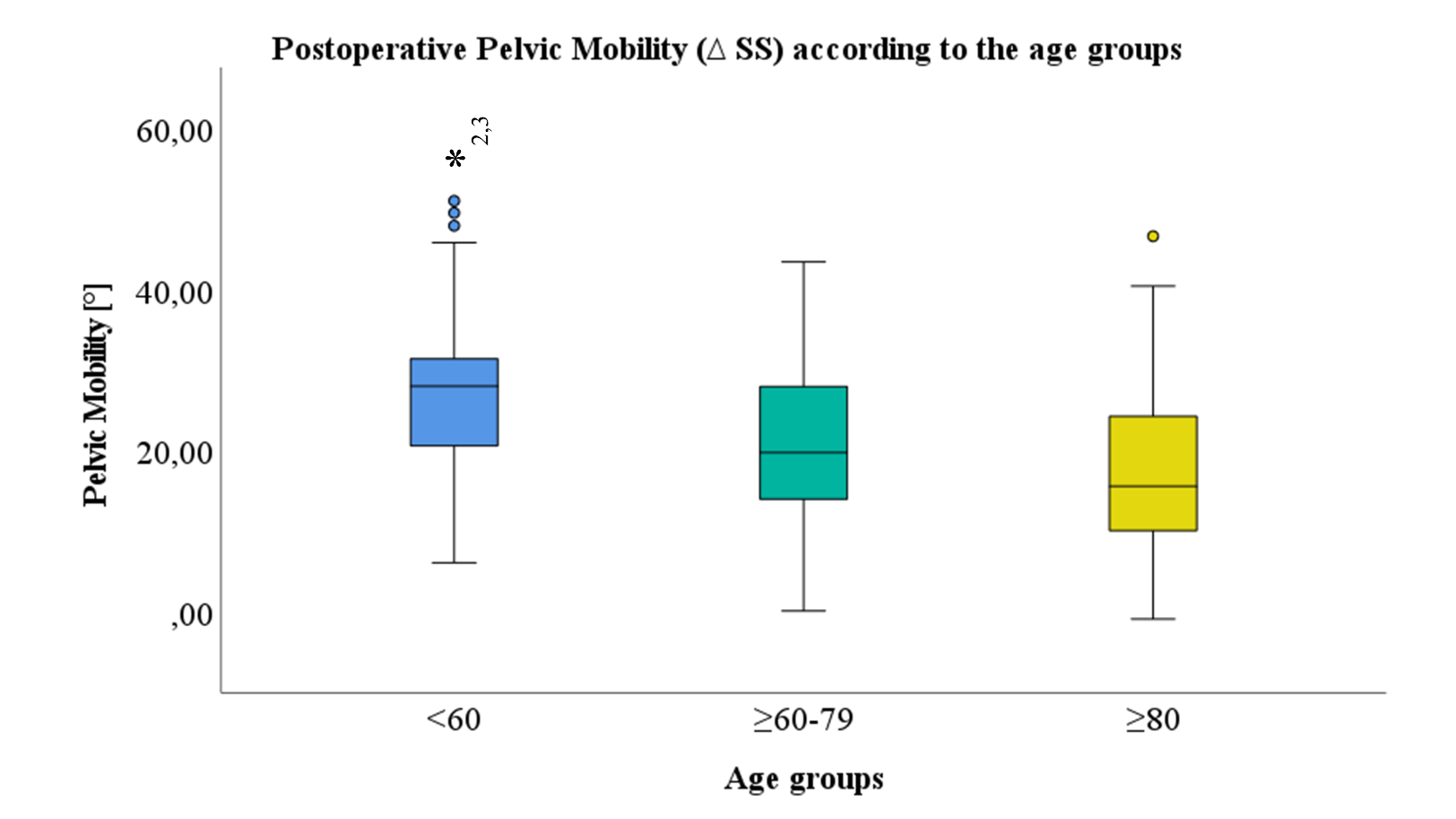


**Supplement figure 2.** Postoperative pelvic mobility (∆SS) as a key segment of the spinopelvic complex is depicted in relation to the defined age: group 1 < 60 years, group 2 ≥60-79 years and group 3 ≥80 years. *_2,3_ indicating significant differences between group 1 and group 2 and 3 respectively.


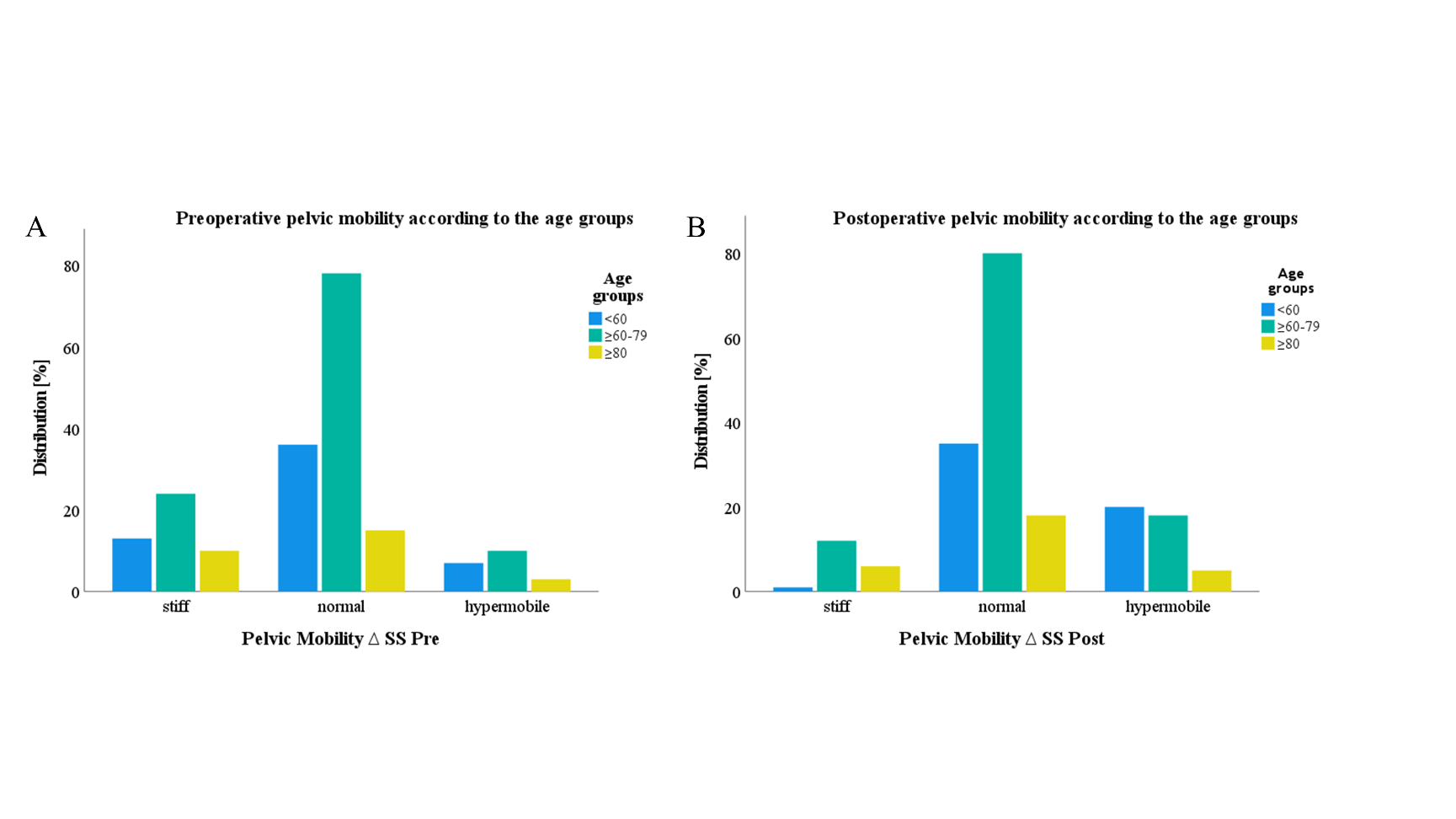


**Supplement figure 3.** Distribution of stiff (∆ SS<10°), normal (∆ SS≥10°-30°), and hypermobile (∆ SS>30°) pelvic mobility based on ∆ SS=SS_standing_-SS_sitting_ according to the defined age groups pre- (A) and postoperatively (B).


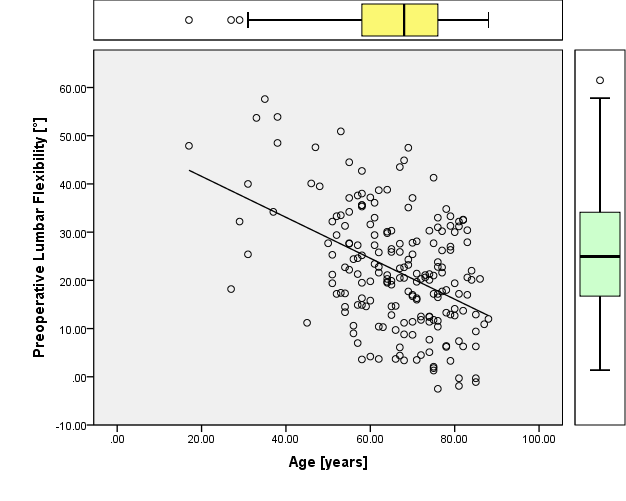
 **Supplement figure 4.** Linear regression analysis depicting age and preoperative lumbar flexiblity (∆ LL). The analyses was performed with Pearson correlation coefficient (r=-0.449, p<0.000).


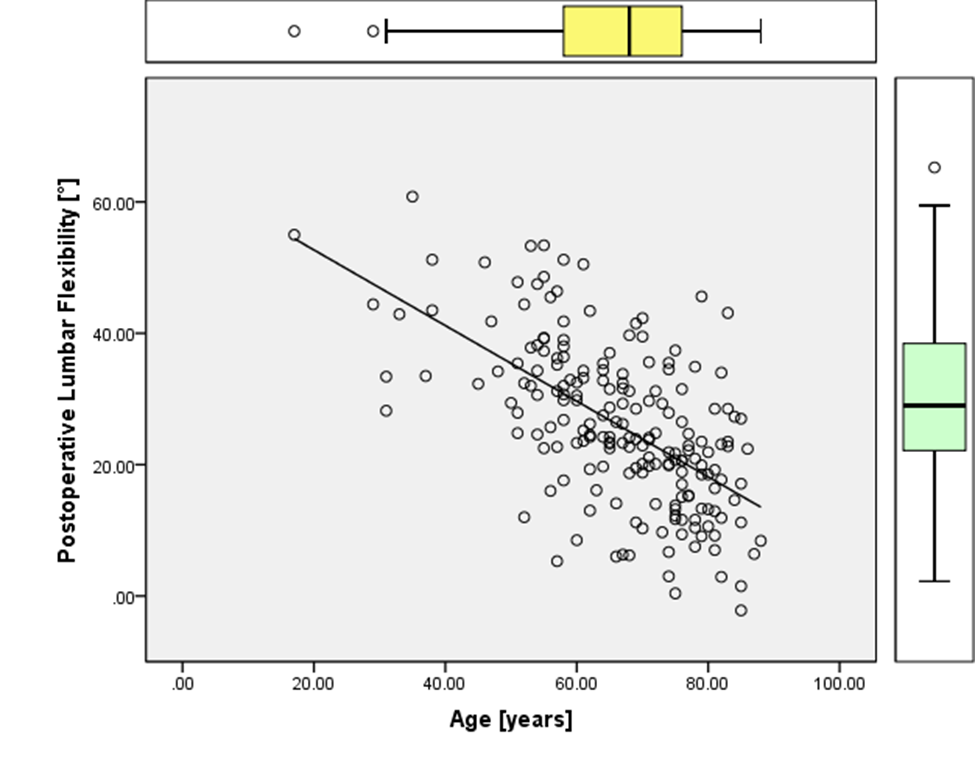
 **Supplement figure 5.** Linear regression analysis depicting age and postoperative lumbar flexiblity (∆ LL). The analyses was performed with Pearson correlation coefficient (r=-0.597, p<0.000).

**Components and Fixation Techniques**

| **Component / Fixation** | **Manufacturer** | **Number (N)** |
| --- | --- | --- |
| Cementless Fixation Technique |  | 197 |
| Allofit Cup | Zimmer Biomet, Warsaw, USA | 193 |
| TMT Cup | Zimmer Biomet, Warsaw, USA | 1 |
| R3 Cup | Smith & Nephew, London, UK | 3 |
| UHMWPE- Inlay | depends on the other components | 194 |
| Ceramic- Inlay | depends on the other components | 3 |
| Ceramic Head | Zimmer Biomet or Smith & Nephew manufactured by Biolox Delta, CeramTec, Plochingen, Germany | 197 |
| SL-Plus MIA Standard Offset Stem | Smith & Nephew, London, UK | 114 |
| SL-Plus MIA Lateral Offset Stem | Smith & Nephew, London, UK | 15 |
| SLR-Plus Stem | Smith & Nephew, London, UK | 1 |
| Avenir Standard Offset Stem | Zimmer Biomet, Warsaw, USA | 14 |
| Avenir Lateral Offset Stem | Zimmer Biomet, Warsaw, USA | 1 |
| Avenir Complete Standard Offset Stem | Zimmer Biomet, Warsaw, USA | 42 |
| Avenir Complete High Offset/  Coxa Vara Stem | Zimmer Biomet, Warsaw, USA | 10 |

**Supplement Table 1.** Overview of the fixation techniques applied the components and manufacturers used, and their quantities.

**Radiological Parameter**

| **Radiological Parameter** | **Description** |
| --- | --- |
| C7-Sagittal vertical axis (C7-SVA) | Horizontal distance between a line from the center of the C7 vertebral body to the posterior superior corner of the sacral endplate and a plumb line from the center of the C7 vertebral body. |
| Cervical lordosis (CL) | Angle between inferior endplate of C2 and inferior endplate of C7 |
| Thoracic kyphosis (TK) | Angle between upper vertebral body marking the beginning of thoracic curve and the inferior border of the vertebral body at the interface between thoracic and lumbar curves. |
| Lumbar lordosis (LL) | Angle between superior endplate of L1 and superior endplate of S1. |
| Pelvic incidence (PI) | Angle between the line connecting the midpoint of the superior plate of S1 and the midpoint of the hip axis with the line perpendicular to the superior plate of S1. |
| Anterior plane pelvic tilt (APPT) | Angle between the line connecting the midpoint of both anterior superior iliac spines to the pubic symphysis, and a vertical line. |
| Sacral slope (SS) | Angle between the superior endplate of S1 and a horizontal line. |
| Pelvic femoral angle (PFA) | Angle between the center of the hip axis to the midpoint of the superior sacral endplate and a 10 cm line from the center of the hip axis to the ventral cortex of the femur |

**Supplement Table 2**. Measured radiological parameters with description arranged by global sagittal spinal alignment, sagittal spinal parameter, and spinopelvic parameter.

**Interrater reliability**

|  | *Preoperative* | *Postoperative* | *Mean (Pre-postoperative)* | |  |
| --- | --- | --- | --- | --- | --- |
| C7-Sagittal vertical axis | .614 | .571 | | .593 | |
| Cervical lordosis | .662 | .691 | | .677 | |
| Thoracic kyphosis | .652 | .638 | | .645 | |
| Lumbar lordosis | .746 | .779 | | .763 | |
| Pelvic incidence | .534 | .728 | | .631 | |
| Sacral slope | .533 | .711 | | .622 | |
| Anterior plane pelvic tilt | .520 | .554 | | .537 | |
| Pelvic femoral angle | .694 | .831 | | .763 | |

**Supplement Table 3.** Pre-and postoperative values and the mean of interrater reliability of the the global spinal alignment, spinal sagittal and spinopelvic parameter. Spearman´s rank correlation coefficient was used.

| Pre Mobility (∆ SS) | Post Mobility (∆ SS) | <60 years | ≥60-79 years | ≥80 years |
| --- | --- | --- | --- | --- |
| Stiff | Stiff | 1.8% (1) | 4.5% (5) | 13.8% (4) |
|  | Normal | 16.1% (9) | 14.3% (16) | 20.7% (6) |
|  | Hypermobile | 5.4% (3) | 2.7% (3) | 0% (0) |
| Normal | Stiff | 0% (0) | 7.1% (8) | 6.9% (2) |
|  | Normal | 42.8% (24) | 54.5% (61) | 34.5% (10) |
|  | Hypermobile | 21.4% (12) | 8.0% (9) | 10.3% (3) |
| Hypermobile | Stiff | 0% (0) | 0% (0) | 0% (0) |
|  | Normal | 3.6% (2) | 2.7% (3) | 6.9% (2) |
|  | Hypermobile | 8.9% (5) | 6.2% (7) | 6.9% (2) |

**Supplement Table 4.** Patient flow in terms of their preoperative and postoperative classification of pelvic mobility based on ∆ SS=SS_standing_-SS_sitting_ defined as stiff (∆ SS<10°), normal (∆ SS≥10°-30°), and hypermobile (∆ SS>30°) according to the age: group 1 < 60 years, group 2 ≥60-79 years and group 3 ≥80 years. The results are presented as percentage % and total number(N).

| **Age [years]** | **Preoperative** | r | p-value | **Postoperative** | r | p-value |
| --- | --- | --- | --- | --- | --- | --- |
| Lumbar Flexibility (∆LL) |  | -0.449 | 0.000 |  | -0.597 | 0.000 |
| Pelvic Mobility (∆SS) |  | -0.151 | 0.035 |  | -0.394 | 0.000 |
| Hip Motion (∆PFA) |  | 0.047 | 0.512 |  | 0.190 | 0.008 |

**Supplement table 5.** Linear regression analysis depicting age and preoperative and postoperative parameter of spinopelvic mobility. The analyses was performed with Pearson correlation coefficient.
